# Optimizing treatment for the prevention of pre-eclampsia/eclampsia in Nepal: is calcium supplementation during pregnancy cost-effective?

**DOI:** 10.1186/s12962-016-0062-3

**Published:** 2016-12-28

**Authors:** Isabelle Feldhaus, Amnesty E. LeFevre, Chandra Rai, Jona Bhattarai, Deirdre Russo, Barbara Rawlins, Pushpa Chaudhary, Kusum Thapa

**Affiliations:** 1 Department of International Health, Johns Hopkins Bloomberg School of Public Health, Johns Hopkins University, 615 N. Wolfe Street, Baltimore, MD 21205 USA; 2 Jhpiego, Oasis Building, Patan Dhoka, Lalitpur, Nepal; 3 Jhpiego, 615 Thames Street, St# 200, Baltimore, MD 21231 USA; 4 Maternal and Child Health Integrated Program, Jhpiego, 1776 Massachusetts Avenue, NW, Washington, DC 20036 USA; 5 Ministry of Health, Government of Nepal, Teku, Kathmandu Nepal

**Keywords:** Pre-eclampsia, Eclampsia, Maternal mortality, Nepal, Low-income countries, Antenatal care, Calcium, Micronutrients, Magnesium sulfate, Cost-effectiveness

## Abstract

**Background:**

In Nepal, pre-eclampsia/eclampsia (PE/E) causes an estimated 21% of maternal deaths annually and contributes to adverse neonatal birth outcomes. Calcium supplementation has been shown to reduce the risk of PE/E for pregnant women and preterm birth. This study presents findings from a cost-effectiveness analysis of a pilot project, which provided calcium supplementation through the public sector to pregnant women during antenatal care for PE/E prevention as compared to existing PE/E management in Nepal.

**Methods:**

Economic costs were assessed from program and societal perspectives for the May 2012 to August 2013 analytic time horizon, drawing from implementing partner financial records and the literature. Effects were calculated as disability-adjusted life years (DALYs) averted for mothers and newborns. A decision tree was used to model the cost-effectiveness of three strategies delivered through the public sector: (i) calcium supplementation in addition to the existing standard of care (MgSO_4_); (ii) standard of care, and (iii) no treatment. Uncertainty was assessed using one-way and probabilistic sensitivity analyses in TreeAge Pro.

**Results:**

The costs to start-up calcium introduction in addition to MgSO_4_ were $44,804, while the costs to support ongoing program implementation were $72,852. Collectively, these values correspond to a program cost per person per year of $0.44. The calcium program corresponded to a societal cost per DALY averted of $25.33 ($25.22–29.50) when compared against MgSO_4_ treatment. Primary cost drivers included rate for facility delivery, costs associated with hospitalization, and the probability of developing PE/E. The addition of calcium to the standard of care corresponds to slight increases in effect and cost, and has a 84% probability of cost-effectiveness above a WTP threshold of $40 USD when compared to the standard of care alone.

**Conclusions:**

Calcium supplementation for pregnant mothers for prevention of PE/E provided with MgSO_4_ for treatment holds promise for the cost-effective reduction of maternal and neonatal morbidity and mortality associated with PE/E. The findings of this study compare favorably with other low-cost, high priority interventions recommended for South Asia. Additional research is recommended to improve the rigor of evidence available on the treatment strategies and health outcomes.

## Background

Maternal and neonatal morbidity and mortality due to preventable causes contribute to a staggering proportion of the world’s burden of disease. In 2013, an estimated 289,000 women died from complications associated with pregnancy or childbirth; 27.1% due to hemorrhage, 14.0% hypertensive disorders, and 10.7% sepsis between 2003 and 2009 [1, 2]. Among children under five, 45% of deaths in 2015 occurred within the first 28 days of life with 28% of deaths due to pre-term causes [3–5]. In Nepal, an estimated 1500 maternal and 12,000 neonatal deaths occurred in 2015 [6, 7]. The country has seen reductions in maternal and under-five mortality by 76% and 71%, respectively, since 1990 [7, 8]. However, maternal deaths still make up nearly 10% of deaths among women of reproductive age and surveys show that the reduction in neonatal mortality, at only 34% in Nepal, greatly lags behind achievements in under-five and infant mortality [5, 8].

Eclampsia is the leading direct cause of maternal mortality in Nepal, occurring in 1 in 25 women and resulting in an estimated 21% of maternal deaths annually [9, 10]. The condition is also associated with adverse neonatal outcomes, including higher rates of neonatal intensive care unit admission and length of stay, small for gestational age, stillbirth, and mortality [11–15]. Because termination of pregnancy is the definitive management of gestational hypertension and eclampsia, such complications remain the leading cause of provider-initiated preterm delivery globally [16, 17]. Magnesium sulfate (MgSO_4_) may significantly reduce the risk of recurring seizures to as low as 19%, and is used regularly across Nepal [10, 18–23]. In some cases, cesarean sections are performed to ensure the safety of both mother and child [24]. Efforts toward the prevention of pre-eclampsia/eclampsia (PE/E) may contribute to reductions in maternal and perinatal mortality. In multiple clinical trials, low-dose calcium supplementation has been shown to reduce the risk of PE/E for pregnant women and preterm birth [14, 15, 22, 25, 26]. The World Health Organization (WHO) currently recommends that 1.5–2.0 g of calcium be taken daily during pregnancy for the prevention of PE/E, beginning at a gestational age of approximately 20 weeks [27].

Screening and early identification of women at risk for PE/E may enable appropriate antenatal care (ANC), management, and treatment. Evidence from high resource settings on the cost-effectiveness of PE/E screening is favorable [28, 29]. Findings from 33 countries included in the Magnesium Sulphate for Prevention of Eclampsia (Magpie) Trial suggest that prophylactic treatment of PE/E with MgSO_4_ is cost-effective for severe cases and when magnesium sulfate is available at little to no cost [30]. Analysis of prophylaxis strategies in the United States determined that universal prophylaxis with MgSO_4_ for all women with pre-eclampsia is cost-effective compared with treating only those with severe disease [31]. Meta-analyses assessing the cost-effectiveness of testing and treatment options for PE/E from the perspective of the United Kingdom’s National Health System suggest that providing calcium to all women without initial screening for PE/E is the most effective “test/treatment” option [29, 32]. However, the existing literature fails to conclusively determine whether calcium supplementation interventions are cost-effective in low-income settings.

From May 2012 through August 2013, the Ministry of Health and Population (MOHP) in Nepal, with technical support from the USAID-funded Maternal and Child Health Integrated Program (MCHIP) and Jhpiego, implemented a pilot program testing the feasibility of providing calcium supplements to pregnant women to prevent PE/E. The objective of the overarching study was to evaluate this pilot program and generate evidence informing future decisions for scale up. The primary aim of this model-based sub-study is to determine the incremental cost-effectiveness of calcium supplementation for PE/E prevention as compared to existing curative PE/E management in Nepal. Secondary objectives sought to explore the incremental cost effectiveness of three alternatives: (i) calcium supplementation in addition to MgSO_4_ (i.e. the existing standard of care), (ii) the existing standard of care, and (iii) no treatment for PE/E.

Ethical approval was obtained from Nepal Health Research Council and the Johns Hopkins University Bloomberg School of Public Health institutional review board.

## Methods

### Study setting and context

This pilot program was implemented in Dailekh District in the Mid-Western Region of Nepal based on the recommendations of a Technical Advisory Group formed to guide program implementation. Dailekh District, 650 km from Kathmandu, covers 1505 km^2^ with elevation ranges from 544 to 4168 m above sea level [33]. This mountainous district was chosen because its terrain represents the most common type of district in Nepal while having a greater population. As a result, it may most accurately illustrate the challenges associated with implementation nationwide. Dailekh had an estimated population of 264,616 in 2011, 51% of whom are women [33, 34].

The District Health Office provides preventive, curative, and promotional health services through 60 Health Facilities (HFs) [33]. Over 800 female community health volunteers (FCHVs) promote maternal and child health services across the district, corresponding to 1 FCHV per 1000 population. Utilization rates for key reproductive, maternal, newborn and child health services exceed national level estimates for most critical indicators. During pregnancy, 29% of women report receiving no ANC services at all compared to a national rate of 15.2% [33, 35]. Similar trends are reported for delivery careseeking with 59% of women delivering with the assistance of a skilled birth attendant, compared to 36% of women nationally [33, 35].

### Program

Project activities began in May 2012 and spanned through August 2013. Startup activities included the development of training manuals on calcium supplementation, behavior change communication (BCC) materials, monitoring and evaluation tools, and initiation of training activities. Monitoring and evaluation tools were developed in line with the existing health management information system tools (i.e. calcium registers for ANC providers, calcium information added to existing FCHV registers, and reporting forms for village health workers and facility-level health providers). The supply of calcium supplements was purchased from Missionpharma India in 2012 and from Curex Pharmaceuticals Nepal in 2013.

To initiate district level activities, a half-day orientation was held for district level stakeholders. Four one-day training of district-level trainers with 95 attendees total followed. Sixty-one-day trainings at the health facility level trained all 268 ANC health workers in the district, while another 60 one-day trainings trained all 810 FCHVs. A full calcium supply and logistics support was provided to all health facilities. ANC health workers at government health facilities were trained to counsel pregnant women on the benefits of calcium supplementation during pregnancy and to distribute calcium during ANC visits. Health workers were also trained in the use of magnesium sulfate for treatment of severe PE/E and to perform PE/E screening using blood pressure measurements and urine protein tests, conducted upon each ANC visit. Health facilities were provided with urine collection bottles and dipsticks to ensure the availability of PE/E screening services for pregnant women during ANC visits. FCHVs were trained to promote ANC attendance and compliance with the recommended calcium regimen. Pregnant women attending an ANC visit were given a calcium supply for the remainder of their pregnancy. Women were typically provided with 300 calcium tablets if enrolled at ≤5 months gestational age and 100–200 tablets if enrolled at later gestational age. The recommended calcium intake was 1 g daily (2 tablets of 500 mg each), beginning at 16 weeks gestational age until delivery.

### Comparators

The national standard drug for treatment of severe PE/E in Nepal is MgSO_4_, administered by health facility providers following screening for PE/E [23]. Program activities sought to expand the existing standard of care to include the provision of calcium supplements (i.e. MgSO_4_ + Calcium). In this analysis, we compare the costs and effects of the (i) MOHP calcium supplementation program in addition to the standard of care (i.e. MgSO_4_ + Calcium) against two hypothetical arms, (ii) MgSO_4_ treatment alone (i.e. standard of care in Nepal) and (iii) no treatment.

### Calculating costs

Economic costs, presented here in 2014 US dollars (USD), were assessed using a societal perspective, inclusive of incremental costs to users and the government and/or health system. Program costs were obtained from financial records provided by implementing partners (MCHIP and Jhpiego), and adjusted according to the 2014 consumer price indices and market exchange rates. An annualized factor comprised of a 3% discount rate and WHO-CHOICE life expectancy estimates were used to annualize capital costs [36]. Program costs included capital (equipment, supplies, initial trainings), and recurrent (supervision, personnel costs, calcium supplements and MgSO_4_ treatment, PE/E screening supplies, and IEC materials) costs required to start-up and support ongoing implementation [21]. While the pilot program experienced calcium supply costs from two sources as a result of supply shortage during implementation, modeled costs were calculated assuming no future shortfall in calcium supply. To estimate costs to users and incremental costs to health system, additional data were drawn from the literature and a survey implemented by Jhpiego in three large government hospitals (Table 1). Based on survey findings, individual costs to hospitalize mothers, newborns, and PE/E cases were obtained, including costs of inpatient bed days, transportation, hospital personnel, cesarean section procedures, and other services.

**Table 1:** Total costs and inputs in 2014 USD

Indicator	Value	Distribution for PSA
Total fixed program costs		
Start-up costs		
Capital	$33,884.48	–
Recurrent costs	$10,919.61	–
Total	$44,804.09	–
Implementation costs		
Recurrent costs	$72,827.94	–
Blood pressure instrument	$24.25	–
Total (not incl. variable costs, listed below)	$72,852.19	–
Total program costs (per individual)	$0.44	
Variable costs per individual		
Urine test (sample bottle + dipstick)	$0.07	Lognormal
Calcium supply (per bottle, 100 tablets)	$0.69	Lognormal
MgSO_4_ treatment regimen	$13.00 [21]	Lognormal
Mean daily bed fee for maternal hospitalization	$4.21	Lognormal
Other maternal hospitalization costs	$31.66	Lognormal
Mean daily bed fee for newborn hospitalization	$8.25	Lognormal
Other newborn hospitalization costs	$21.11	Lognormal
Mean indirect costs per individual		
Daily wage in district	$3.36 [42]	Lognormal
Round trip transportation for hospitalization	$29.67 [41]	Lognormal
Mean duration of hospital stay		
Normal, healthy case		
Vaginal birth	1 day	–
Cesarean section	4.3 days	–
Live newborn	1 day	–
PE/E case		
Vaginal birth	6 days	–
Cesarean section	10 days	–
Live newborn	7 days	–

Source: Jhpiego/MCHIP

### Calculating effectiveness

Effects were calculated as disability-adjusted life years (DALYs) drawing from primary and secondary sources [37]. Estimates of the Years of Life Lost (YLLs) both to mothers and newborns were drawn from peer-reviewed literature, Nepal Demographic and Health Survey 2011, the Nepal Maternal Mortality and Morbidity Study 2008/2009, and primary data collected by Jhpiego on the calcium supplementation program (Table 2). Resulting probabilities for maternal and newborn mortality were adjusted according to the treatment received via each pathway through the model. Resulting effects were standardized to one million population.

**Table 2:** Background epidemiological and program data

Parameter	Base case	Low	High	Distribution for PSA
Maternal careseeking				
ANC, at least one visit	84.8% [35]	63.60%	100%	Beta
Facility deliveries in government sector	26.0% [35]	19.5%	32.5%	Beta
Screening procedures				
Blood pressure	86.4% [35]	64.80%	100%	Beta
Urine sample	55.9% [35]	41.9%	69.9%	Beta
Blood sample	45.3% [35]	34.0%	56.63%	Beta
Eclampsia epidemiology				
Incidence of eclampsia	4.30% [9]	3.23%	5.38%	Beta
Eclampsia as direct maternal cause of death	21.0% [10]	15.8%	26.3%	Beta
Mean age of eclampsia patients	23.4 [38]	17.7 [38]	29.2 [38]	–
Treatment				
MgSO4 for PE/E management	68.9%^a^	51.7%	95.8%	Beta
Cesarean delivery	55.31% [46]	41.5%	69.1%	Beta
Maternal risk ratios for PE/E				
Calcium supplementation	0.45 [15]	0.31 [15]	0.65 [15]	Beta
MgSO4, standard regimen	0.19 [19]	0.14 [19]	0.24 [19]	Beta
Cesarean delivery	0.55 [25]	0.41 [25]	0.69 [25]	Beta
Still birth rates				
Vaginal birth	1.37%^a^	1.03%	1.71%	Beta
Cesarean delivery	0.43%^a^	0.32%	0.54%	Beta
Vaginal birth among PE/E cases	14.3%^a^	10.7%	17.9%	Beta
Cesarean delivery among PE/E cases	9.77%^a^	7.33%	12.2%	Beta
Disability-adjusted life years (DALYs)				
DALYs averted per individual (maternal)	24.9^b^	15.6^c^	31.8^d^	–
DALYs averted per individual (newborn)	29.0^b^	16.4^c^	32.3^d^	–
Pilot Program Data, Dailekh District, 2013				
Maternal careseeking				
ANC, at least one visit	94.6%	71.0%	100%	Beta
Calcium regimen compliance				
Full compliance	67.3%	50.5%	84.1%	–
Partial or low compliance	32.7%	24.5%	40.9%	–
Gestational age among women receiving calcium				
4–5 months (300 calcium tablets)	82.2%	61.7%	95%	–
6–7 months (200 calcium tablets)	13.8%	25.6%	2.5%	–
8–9 months (100 calcium tablets)	4.0%	12.8%	2.5%	–
Screening procedures				
Blood pressure	98%	73.5%	100%	Beta
Urine test	97%	72.8%	100%	Beta

^a^Source: Jhpiego/MCHIP

^b^Discount rate 3% + no age weighting

^c^Discount rate 6% + age weighting

^d^Discount rate 3% + age weighting

The efficacy of MgSO_4_ treatment and cesarean section indicated as risk ratios were applied to outcomes accordingly. Those with partial or low compliance with the full calcium regimen (300 tablets) were conservatively assumed to experience no effects of calcium supplementation in the prevention of PE/E. Because the relevant mortality risk data were unspecified, it was assumed that the probability of maternal and newborn death was similar for at-home and facility deliveries. The average age of a maternal death was 23.4 years based on the mean age of eclampsia patients in Nepal, and the life expectancy for females in Nepal was 69 years of age [38, 39]. Newborn YLLs were determined using the mean life expectancy of males and females in Nepal, which is 68 years of age [39]. To generate years lost to disability (YLDs), disability weights for hypertensive disorders of pregnancy (0.00) [40] were used for mothers. The average age of onset used in the calculation of newborn YLDs was 0.0 years because mortality rates used in the model were associated with stillbirth.

Base case DALYs for mothers and newborns were discounted at a rate of 3% without age weighting. Incremental DALYs averted through the calcium supplementation program were calculated separately for mothers and newborns using relevant parameters, and summed for each study arm to generate a summary estimate of DALYs for each arm.

### Data analysis

**Fig. 1:**
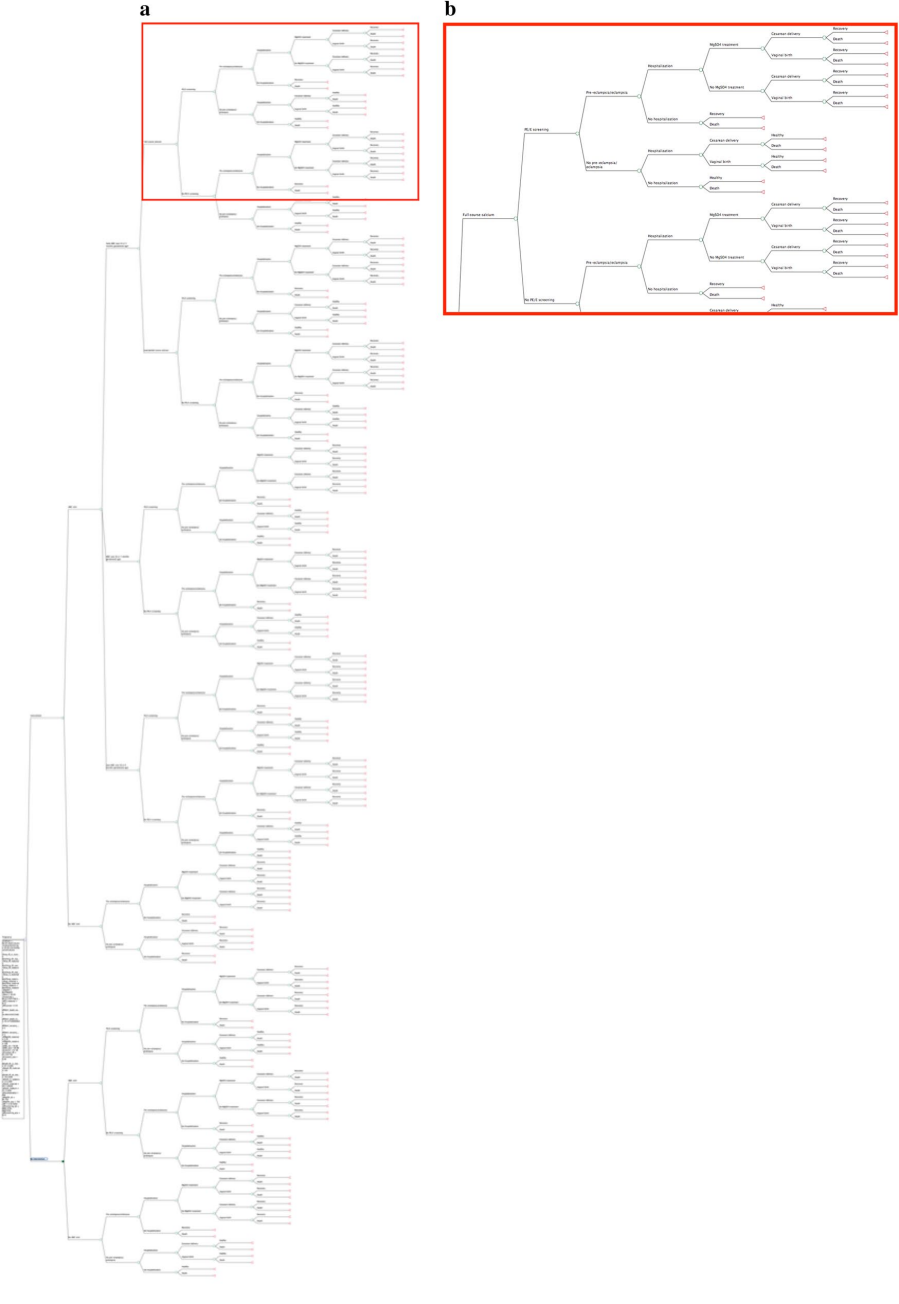
**a** Partial (two comparator arms shown) decision tree model of calcium supplementation program in Nepal. The segment in *red* is magnified. **b** Magnified inset: Decision tree model of calcium supplementation program for pregnant women in Nepal

TreeAge Pro ©2014 was used to generate a decision tree analytic model to conduct incremental cost-effectiveness analyses (Fig. 1). Analyses were built around the possible pathways of a pregnant woman and newborns with respect to PE/E. The tree was divided into three primary branches attributed to each arm of the study: (i) calcium supplementation in addition to MgSO_4_ (i.e. the existing standard of care), (ii) the existing standard of care, and (iii) no treatment for PE/E. For individuals enrolled to receive calcium supplementation in addition to MgSO_4_, additional branches were included for attending at least one ANC visit, coverage of calcium distribution (i.e. gestational age at time of ANC visit), reported compliance with the full course of calcium, PE/E screening, onset of PE/E, hospitalization, MgSO_4_ treatment, cesarean section, and final health status of mothers and newborns (i.e. healthy/recovery or death). For the comparator arms reflecting (ii) existing standard of care and (iii) no treatment, similar sub-branches were used with the exception of those associated with calcium supplementation and MgSO_4_, as appropriate. The model represented a 1-year analytic time horizon consistent with the duration of the pilot program.

Comparisons of costs and effects for each study arm were used to generate incremental cost effectiveness ratios (ICERs). Variations in individual parameters were tested within this framework using one-way and multi-way sensitivity analyses. The probabilities of compliance with the calcium regimen, risk ratio for the prevention of PE/E with calcium supplementation, and rates of screening, hospitalization, MgSO_4_ treatment, and cesarean section were varied. The costs associated with calcium supply, magnesium sulfate treatment, urine tests, and blood pressure testing were highlighted as costs of interest in these analyses as these were key commodities used in implementation of the program. These costs of interest as well as costs related to hospitalization were varied by 25% in sensitivity analyses with the exception of calcium supply, which had an upper bound of $1.42 USD per 100 tablets based on supplier prices (Table 1). Effects were varied from a base case of 3% discounting and no age weighting, to a high value under 3% discounting and age weighting and a low of 6% discounting and age weighting. A tornado diagram was generated to display variations in individual parameters in the univariate sensitivity analysis. Probabilistic sensitivity analyses explored the effect of simultaneous variations in multiple parameters.

### Thresholds for determining cost-effectiveness

Overall findings on the cost per DALY averted for each option were evaluated according thresholds established in 2002 by the Commission on Macroeconomic and Health (CMH).^1^ Findings were additionally contextualized against estimates of the cost per DALY averted for other low cost high priority interventions stipulated by *Disease Control Priorities, 2nd ed.* and other examples of calcium and MgSO_4_ programs published in the literature. Cost-effectiveness acceptability curves were generated to approximate the proportion of iterations that were cost-effective for each study arm for willingness-to-pay (WTP) thresholds between $0 and $100 USD.

## Results

### Costs

Table 1 presents input parameters used to generate cost estimates. Start-up costs to provide calcium in addition to the existing standard of care were $44,804 and 1-year implementation costs were $72,852; these values correspond to a program cost per person per year of $0.44. Costs to users, which included transportation and daily wages lost, were $29.67 [41] and $3.36 [42], respectively. Variable costs for patient care included screening ($0.07), calcium supply ($0.69 per 100 tablets), the total cost of medicines and procedures associated with MgSO_4_ treatment ($13.00) [21], and daily bed fees for mothers ($4.21) and newborns ($8.25). Table 3 outlines the total costs associated with each condition considered in the model. Total inpatient costs to the health system were calculated according to the mean duration of hospital stay by health condition. The average duration of hospital stay for PE/E cases was estimated to be six days, and for those undergoing cesarean section, ten days. Newborns delivered by PE/E mothers remained hospitalized for an average of 7 days.

**Table 3:** Summary of total costs and effects by comparator

Condition	Total costs	Total effects
No treatment	$26.82	52.58
MgSO4	$26.98	52.68
MgSO4 + calcium	$29.29	52.71

### Effects

All pregnant women attending at least one ANC visit were provided with a calcium supply corresponding to gestational age. ANC users at 4 or 5 months of pregnancy (82.2%) received 300 tablets, at 6 or 7 months (13.8%) received 200 tablets, and at 8 or 9 months (4.0%) received 100 tablets (Table 2). Of those women receiving calcium supplements, 67.3% reported adherence to the full course. The remaining 32.7% reported either completing a partial course or low compliance with the calcium regimen. Among women in the pilot program, the added probability of getting screened for PE/E risk through blood pressure measurement or urine testing was high (99.94%). The average risk ratio of PE/E with calcium supplementation during pregnancy is 0.45 (95% CI: 0.31, 0.65) [15]. This efficacy was applied in the model only for those women receiving and compliant with the full course (300 tablets) of calcium supplementation.

The proportion of births delivered in government facilities nationally (26%) was used as a proxy measure for the rate of hospitalization in Nepal since these data were not readily available, either nationally or from program data [35]. Among surveyed government hospitals, 68.9% of women received m MgSO_4_ treatment for severe PE/E management. This value was used to indicate the proportion of women receiving MgSO_4_ sulfate treatment. The efficacy of MgSO_4_ in the treatment of PE/E used in the analysis was a risk ratio of 0.19 [19]. The probability of a cesarean section for inpatients of government hospitals was determined by the rate of cesarean sections as a mode of delivery (55.3%) and their availability in government hospitals (71%) [10, 19]. The mortality rate used for pregnant women in the model was specific to eclampsia as a direct cause of maternal death (21%) [10]. Table 3 indicates the total effects associated with each condition considered in the model.

### Incremental cost-effectiveness

**Fig. 2:**
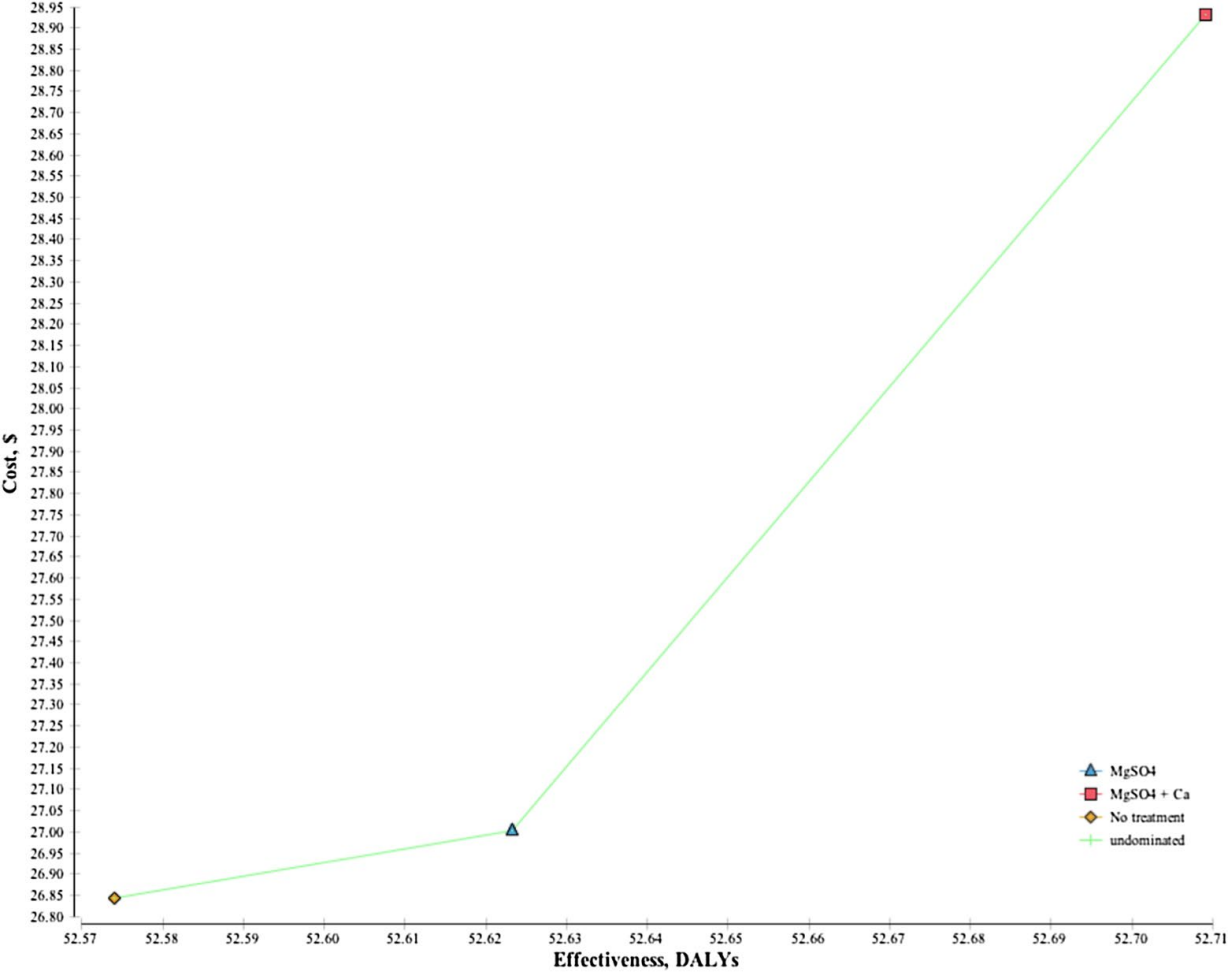
Cost-effectiveness plane showing mean estimates for 10,000 iterations of total costs and effects

**Fig. 3:**
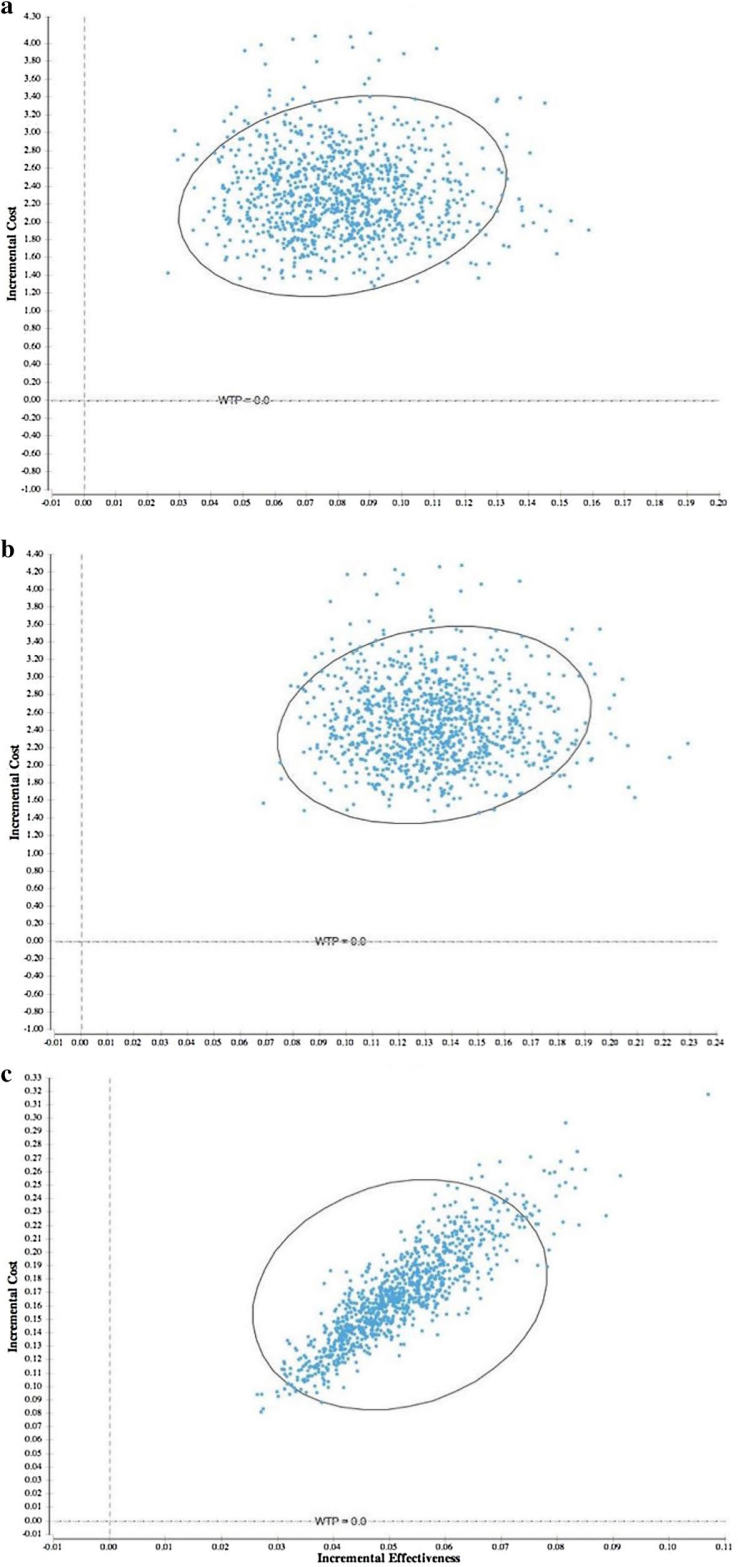
Incremental cost-effectiveness scatter plot for comparison of three alternatives. *Gray line* indicates 95% confidence interval. **a** (i) calcium program vs. (ii) standard of care; **b** (i) calcium program vs. (iii) no treatment; **c** (ii) standard of care vs. (iii) no treatment

Table 4 presents incremental cost-effectiveness ratios for MgSO_4_ vs. no treatment; MgSO_4_ + calcium vs. no treatment; and MgSO_4_ + calcium vs. MgSO_4_. Figure 2 depicts the cost-effectiveness plane showing mean estimates for 10,000 iterations of total costs and effects, while Fig. 3 present incremental cost-effectiveness scatter plots for each alternative considered. MgSO_4_ vs. no treatment was associated with a cost per DALY averted of $3.40 ($2.60–4.00) USD. MgSO_4_ + calcium vs. no treatment corresponded to a cost per DALY averted of $17.50 ($17.14–19.69) USD. When compared to the existing standard of care, the calcium program yields a cost per DALY averted of $25.33 ($25.22–29.50) USD.

**Table 4:** Summary of incremental cost-effectiveness ratios

Comparison	Deterministic calculations			Probabilistic analyses								
	Total incremental costs	Total incremental DALYs averted	Incremental cost per DALY averted	Total incremental costs	Lower bound	Upper bound	Total incremental DALYs averted	Lower bound	Upper bound	Incremental cost per DALY averted	Lower bound	Upper bound
MgSO_4_^a^ vs. no treatment	$0.16	0.05	$3.28	$0.17	$0.13	$0.20	$0.05	$0.05	$0.05	$3.40	$2.60	$4.00
MgSO_4_ + calcium vs. no treatment	$2.47	0.13	$18.47	$2.45	$2.40	$2.56	$0.14	$0.14	$0.05	$17.50	$17.14	$19.69
MgSO_4_ + calcium vs. MgSO_4_^a^	$2.31	0.08	$27.29	$2.28	$2.27	$2.36	$0.09	$0.09	$0.08	$25.33	$25.22	$29.50

^a^Standard of care

**Fig. 4:**
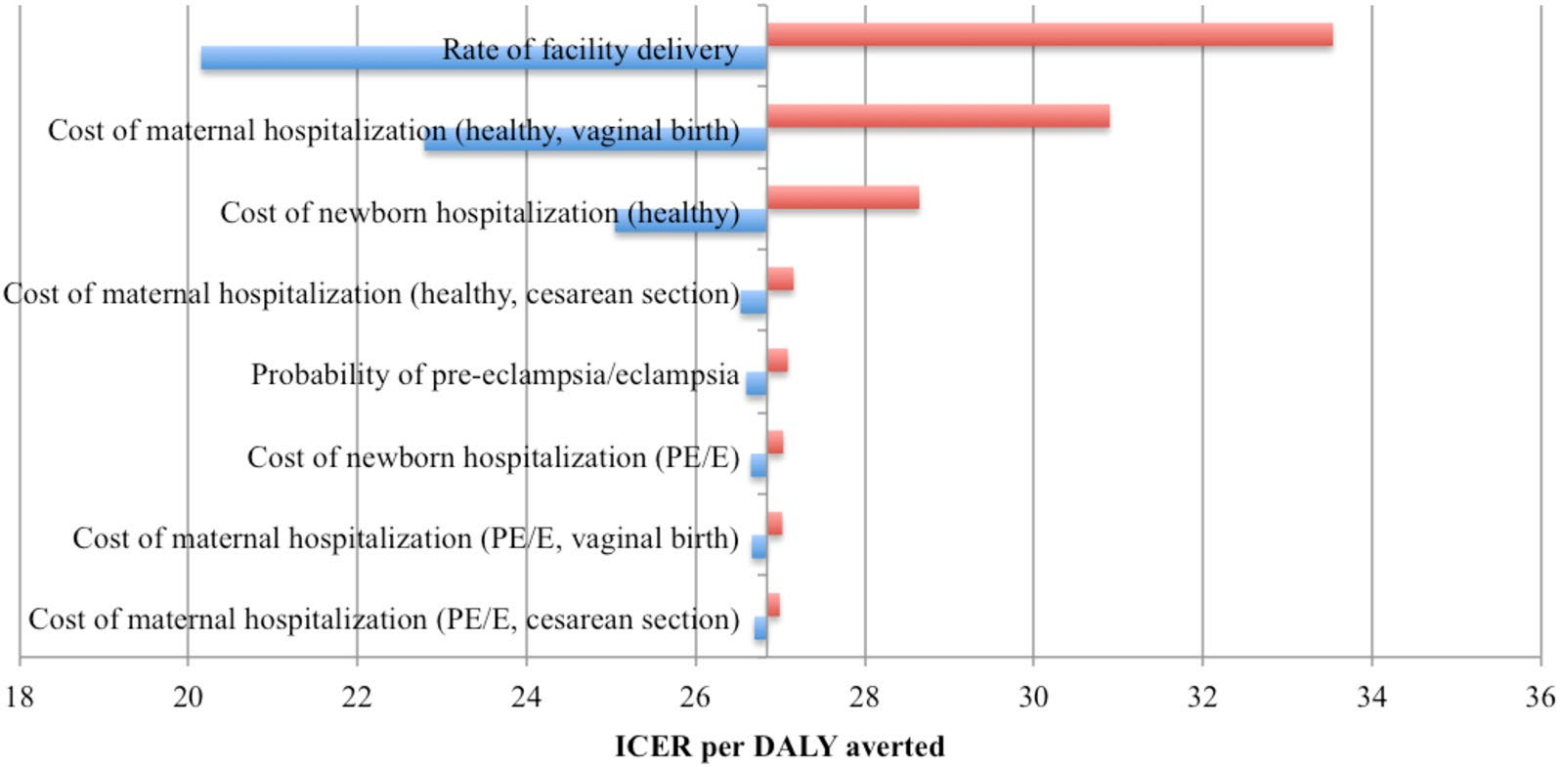
Tornado diagram (net benefits) illustrating the key drivers of cost-effectiveness. The base ICER is set at $26.84 per DALY averted in the diagram. Varying rate of facility delivery resulted in the greatest change in ICER, while varying hospitalization costs for PE/E cases resulted in the least change

**Fig. 5:**
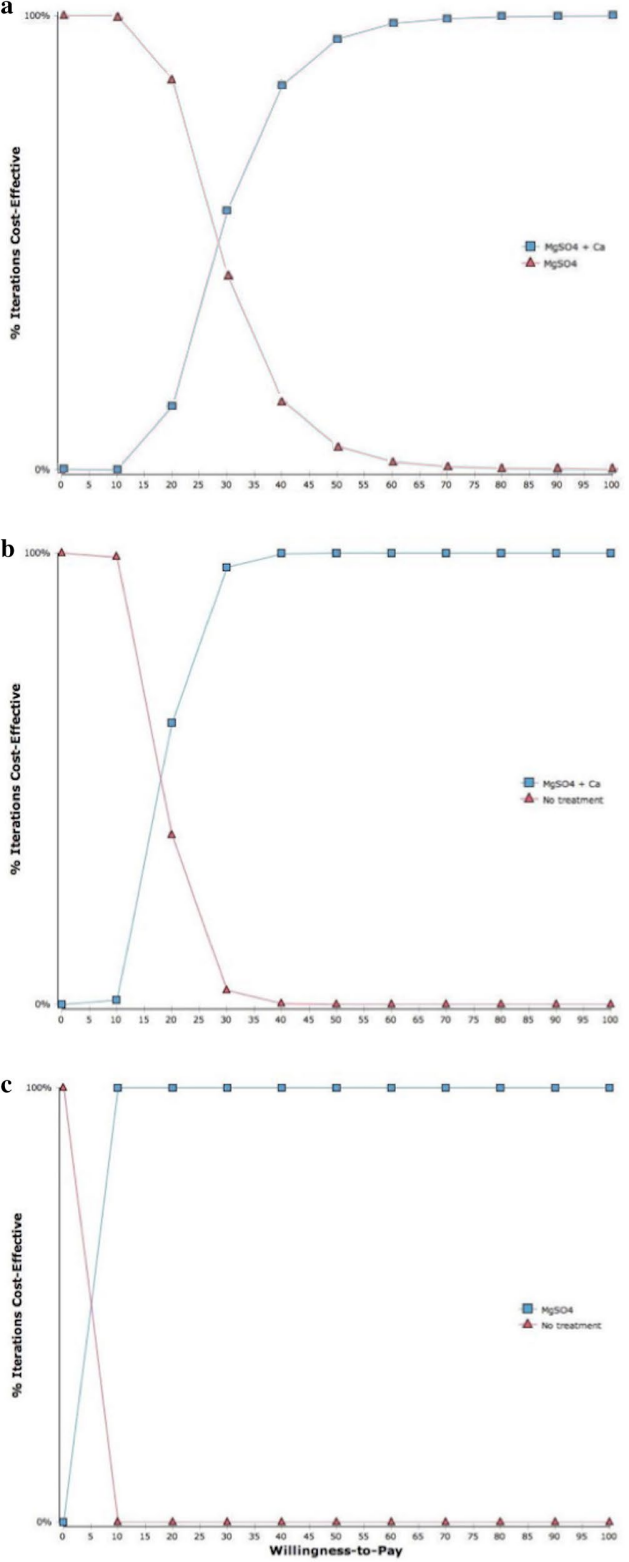
Cost-effectiveness acceptability curves for three alternatives. **a** Calcium program (*blue*) vs. standard of care (*red*); **b** Calcium program (*blue*) vs. no treatment (*red*); **c** Standard of care (*blue*) vs. no treatment (*red*). The curves represent the percentage of iterations that were cost-effective (*y*-axis) for varying willingness-to-pay thresholds in 2014 US$ up to a ceiling $100. Findings suggest that the addition of calcium to the existing standard of care (MGSO4) is favored above a WTP threshold of ~$30 USD. When compared against a scenario of no treatment (B), MgSO4 + calcium is the preferred strategy for WTP thresholds of $25 or more. When considered independently of calcium supplementation, MGSO4 vs. no treatment is considered good value for money above WTP thresholds of $10 USD

One-way sensitivity analyses revealed that incremental cost-effectiveness ratios were driven by four key parameters: (1) hospitalization for facility delivery, (2) cost of hospitalization, (3) cost of cesarean section, and (4) the probability of developing PE/E (Fig. 4). Probabilistic sensitivity analyses simulated the effect of simultaneous variations in multiple iterations (10,000). Findings suggest that the addition of calcium to the existing standard of care (MgSO_4_) is favored above a WTP threshold of ~$30 USD and has an 84% probability of being cost-effective above $40 USD (Fig. 5). When compared against a scenario of no treatment, MgSO_4_ + calcium is the preferred strategy for WTP thresholds of $25 or more. When considered independently of calcium supplementation, MgSO_4_ vs. no treatment is 100% cost-effective above WTP thresholds of $10 USD.

## Discussion

Study findings suggest that MgSO_4_ treatment of PE/E in Nepal is cost-effective when compared to no treatment at all. The addition of calcium supplementation increases costs and corresponds to a minor increase in effectiveness when compared against a scenario of no PE/E management. Despite the low incremental cost-effectiveness ratios across comparisons, findings suggest that for individuals only willing to pay $10 USD or less, MgSO_4_ is the preferred strategy for preventing PE/E. However, for individuals willing to pay more than $35 USD, calcium in addition to MgSO_4_ offers the best value for money.

When compared against alternative resource uses, MgSO_4_ + calcium and MgSO_4_ compare favorably against alternative low-cost high-priority interventions recommended for adoption in South Asia (Table 5). These interventions include increased primary care coverage, improved quality of comprehensive emergency obstetric care, improved overall quality and coverage of care, and neonatal packages targeted to families, communities, and clinics (Table 5). Beyond comparison with other health interventions, we compared study findings with those observed in other economic evaluations of PE/E treatment and prevention programs. In the Magpie Trial determined the incremental cost-effectiveness of prophylactic MgSO_4_ for 9996 women with pre-eclampsia from 33 countries; the incremental cost of preventing one case of eclampsia was $456 in low GNI countries and reserving MgSO_4_ prophylaxis for only severe cases of PE/E reduced this estimate to $263 [30]. Overall, the findings of this study fall well beneath the cost per DALY averted estimates observed in Magpie. However, limitations in available data, including those surrounding maternal and newborn health effects, may contribute to the disparities observed.

**Table 5:** Cost per DALY averted associated with low-cost high-priority interventions recommended for South Asia

Low-cost high-priority interventions recommended for South Asia	Mean cost per DALY averted (USD)
Childhood immunization	$8.00 [47]
Additional coverage of traditional Expanded Program on Immunization	
*Full course calcium supplementation (82.2%) with 67.3% compliance in addition to MgSO*_*4*_ *(68.9%) in Nepal*	*$25.33*
HIV/AIDS	$9–126 [47]
Voluntary counseling and testing	
Peer-based programs targeting at-risk groups	
School-based interventions that disseminate information to students	
Prevention of mother-to-child-transmission with antiretroviral therapy	
Surgical services and emergency care	$6–212 [47]
Surgical ward in a district hospital	
Staffed community ambulance	
Training of lay first responders and volunteer paramedics	
Tuberculosis	$8–263 [47]
Childhood vaccination against endemic TB	
Directly observed short-course chemotherapy	
Isoniazid treatment of epidemic TB	
Management of drug resistance	
Using MgSO_4_ prophylaxis for only severe cases of pre-eclampsia in low GNI countries	$263.00 [30]
Maternal and neonatal care	$127–394 [47]
Increased primary care coverage	
Improved quality of comprehensive emergency obstetric care	
Improved overall quality and coverage of care	
Neonatal packages targeted to families, communities, and clinics	
Incremental cost of preventing one case of eclampsia using MgSO_4_ in low GNI countries	$456.00 [30]

The italicized text denotes the study program in Nepal and aims to contextualize our findings against those of alternative resource uses recommended by the Disease Control Priorities (2nd ed)

Beyond efforts to contextualize study findings with alternative programs and resource uses, we considered findings against the gross domestic product (GDP) of Nepal of $697 per capita in 2014 [44]—the threshold value for cost-effectiveness established by WHO CHOosing Interventions that are Cost Effective (WHO-CHOICE) and CMH [45]. The model demonstrates that calcium supplementation in addition to MgSO_4_ becomes more cost-effective with increased rates of PE/E. This suggests that the intervention would offer the greatest value for money in districts with higher incidence rates of the disease. If calcium supplementation is to be nationally adopted, targeting prevention efforts where incidence of PE/E is particularly high is recommended.

In 2005, the Government of Nepal established the Aama program, or Safe Delivery Incentive Program (SDIP), a demand-side financing scheme providing incentives to women to deliver in health facilities in order to improve mothers’ health outcomes and that of their child. In January 2009, user fees were removed for all types of delivery in government health facilities as well as selected accredited private hospitals. Under the Aama program, cash payments in the amount of NPR 500–1500 ($5.28–15.83 USD) depending on district terrain are made to women to pay for transportation to facilities for delivery [43]. The Aama program also provides incentives to the health facility for deliveries; participating health facilities are reimbursed NPR 1000–1500 ($10.55–15.83 USD) for normal deliveries, NPR 3000 ($31.66 USD) for complicated deliveries, and NPR 7000 ($73.87) for cesarean deliveries [43]. However, our model estimates these costs based on surveys conducted at three government hospitals. Survey findings indicated higher mean costs associated with transportation and hospitalization by type of delivery, thereby providing more conservative estimates of anticipated costs incurred by users and the health system.

The majority of cost-effectiveness drivers are associated with processes tied to hospitalization. Increases in the rates of hospitalization for facility delivery generally resulted in increased costs per DALY averted, suggesting that costs bring about greater impact on cost-effectiveness ratios than the health effects brought about by this intervention. Effectiveness data specific to the nuances of compliance to the calcium regimen and for women of different levels of risk of onset would provide better evidence in this regard.

Hospitalization for facility delivery is directly linked with the probability of receiving MgSO_4_ treatment and/or undergoing a cesarean section for PE/E. The cost of hospitalization and the additional cost of cesarean section procedures constituted the highest cost values incorporated into the model. As a significant proportion of overall costs, it corresponds that these cost factors have a greater impact on incremental cost-effectiveness ratios when varied. Still, with improved utilization of facilities for delivery, it may be possible to decrease the costs of hospitalization and cesarean section procedures and generate overall better health outcomes with PE/E hospitalization and treatment.

Increased use of calcium supplementation for the prevention of hypertensive disorders of pregnancy in the South Asia region may also result in reductions in the cost of calcium supply, further improving cost-effectiveness. A critical factor in determining whether or not mothers receive calcium supplementation and/or early treatment is ANC utilization. The Government of Nepal introduced the 4ANC incentive program in July 2009, providing cash payments to women NPR 400 upon following the ANC protocol of four ANC visits at specific gestation times, institutional delivery, and a postnatal visit [43]. Further increasing ANC and early ANC may serve to further increase use of and adherence to calcium. The combination of these efforts for the prevention of PE/E may culminate into a synergistic effect for optimal cost-effectiveness for Nepal and similar settings.

### Limitations

This analysis was carried out retrospectively as part of the evaluation of a small-scale pilot program in the Dailekh District of Nepal. In the absence of a comparison area, the program is compared against hypothetical comparators of no calcium distribution to women during ANC. As extended observation of person-time spent administering calcium supplementation and counseling was not conducted as part of this pilot program evaluation, the time of health care providers to administer and counsel patients has not been included in this analysis. In the model, utilization of MgSO_4_ and cesarean delivery as a treatment for PE/E and their respective effects remained the same across arms regardless of case severity. Severity can also have considerable implications for morbidity and mortality that affect results. Differences in PE/E management according to case severity were not modeled because limited data exists describing differentiated effects for mothers and newborns based on PE/E severity.

We note that women attending at least one ANC visit may have a greater rate of hospitalization for various reasons. It is also possible that there is a greater hospitalization rate and treatment measures provided for mothers experiencing more severe PE/E. These nuances are not captured in the model due to lack of differentiated data. Beyond these parameters, the model relies upon secondary data to address gaps in primary data on mortality and many of the provider and users’ cost categories. Primary data on reported compliance with calcium regimens provided were drawn from household survey data provided by the program and may be vulnerable to recall bias.

## Conclusions

Calcium supplementation for pregnant mothers provided with appropriate MgSO_4_ treatment holds promise for the cost-effective reduction of maternal and neonatal morbidity and mortality associated with PE/E. As modeled by the MOHP calcium supplementation pilot program in Nepal, such calcium interventions may be similarly cost-effective in other low-income settings. Additional research is recommended to further explore the implications of calcium on newborn outcomes and delivery at scale. Future research is needed to understand factors that may improve early initiation of ANC and increase rates of facility delivery. Efforts to deliver calcium at scale offer the potential to yield even greater cost-effectiveness given the high utilization of ANC—the delivery point of calcium supplementation.

## Abbreviations

ANC: antenatal care
BCC: behavior change communication
Ca: calcium
CMH: Commission on Macroeconomic and Health
DALY: disability-adjusted life year
FCHV: Female Community Health Volunteers
GNI: gross national income
ICER: incremental cost-effectiveness ratio
MCHIP: Maternal and Child Health Integrated Program
MgSO_4_: magnesium sulfate
MOHP: Ministry of Health and Population
NPR: nepalese rupee
PE/E: pre-eclampsia/eclampsia
TAG: Technical Advisory Group
USD: United States dollar
WHO: World Health Organization
YLD: years lost to disability
YLL: years of life lost

## Footnotes

1 The CMH stipulated that an intervention is highly cost effective (HCE) if it has a cost per DALY averted which is lower than the Gross Domestic Product (GDP) per capita. Interventions with a cost per DALY averted of less than 3 times the GDP per capita are deemed *cost effective*, while those with a DALY averted which is 3 times that of the GDP are considered *cost ineffective*. Considering costs according to these standardized thresholds will help to facilitate the interpretation of findings by decision-makers.
